# Pericarditis

**Published:** 1833-07-01

**Authors:** 


1. Mémoires, ou Recherches Anatomico-pathologiques sur diverses Maladies. Par *P. Ch. A. Louis.* A Paris, 1826. —Memoire sur la Péricardite.

2. Clinique Medicale. Deuxième Edition. Par *G. Andral.* —Observations sur les Maladies du Pericarde.

It is to the researches of Louis, published in the Révue Medicale, that we are indebted for the first satisfactory mode of diagnosis of pericarditis, and the additions which have been since made to its pathology and treatment have added but little to the information therein contained. The general similarity of the symptoms caused by diseases of the heart and its appendages —the frequency of the occurrence of the affection which forms the subject of the present paper, in connexion with diseases of the heart itself, or the other thoracic organs—the absence (in some cases) of any very obvious signs of its existence, or what is termed its latent form—and the imperfect examinations made of individuals suffering from the affection, constitute the chief causes of the obscurity which has been considered as belonging to this disease. The works of Morgagni, containing, as they do, some of the first accounts of the existence of such a disease, leave us in doubt as to the mode of its diagnosis; the later examples recorded by Corvisart, Bertin, &c. although much more satisfactory, are still insufficient on the same grounds; and even Laennec, after a minute description of the anatomical characters of the disease, confessed that there was still wanting a diagnostic sign of inflammation of the pericardium; those on which he might place the greatest reliance being sometimes caused by other affections of the heart or its appendages. The attachment, and perhaps too exclusive reliance, of this great pathologist on the evidence afforded by the stethoscope, may have made him overlook, or estimate too lightly, the signs on which the present essays prove that the chief confidence is to be placed.

M. Louis' Essay, re-printed, with some additions to the original one, contains the minute details of some cases which have fallen under his own care, and an examination of those which are recorded in other works; and by a careful induction from which, he has established a certain train of symptoms as characteristic of pericarditis, which may be said, at least in the majority of instances, to render its diagnosis a subject of comparative facility. Subsequently to the publication of his essay, M. Andral has recorded, in his Clinique Medicale, the histories of nine cases of the disease, which it will be seen by a comparative analysis are confirmatory of his views; together with two examples of chronic pericarditis, a subject which has not been separately treated by Louis, unless we may consider the duration of some of his cases as fairly entitling them to such a distinction. The absolutely latent state in which, in some instances, pericarditis has been found to exist (by Andral, Rostan, Dr. Latham, &c.), and when the whole of the morbid phenomena which shewed themselves during life appeared to be attributable to inflammation or ramollissement of the brain, but when, upon examination, the brain appeared to be in a perfectly normal state, and the pericardium exhibited signs of inflammatory action, is of very rare occurrence, and, however inexplicable, should not be allowed to diminish our confidence in those symptoms which are found in the great majority of cases, to be connected with the disease, where examination after death has afforded the means of their confirmation. Is it not probable that, in some instances of this kind, the inflammation which occurs in the pericardium may be of a similar character to that inflammation which is not uncommonly set up in serous membranes, during the last few days or hours of a patient's existence who is suffering from a chronic disease, and in whom no suspicion has been excited of its presence, until it was evidenced by an examination after death?

We commence our notice of this Memoir by an analysis of two cases of pericarditis, without complication, the histories of which are very minutely detailed.

1*st Case.* The affection commenced in a healthy young man, aged 27, by an acute pain in the præcordial and epigastric regions and between the shoulders—palpitations—dyspnœa; the palpitations ceased after three days' duration, the pain continuing more or less acute at intervals; constant oppression, becoming more considerable about the 8th day; pulse exceedingly irregular; beat of the heart dull, distant, and often doubtful; cough and expectoration slight; very evident tumefaction of the thorax at the præcordial region, of considerable extent, over the whole surface of which a dull sound is yielded by percussion, and the respiratory murmur is inaudible; percussion sonorous and respiration perfect over the remainder of the chest. With these symptoms were associated infiltration of the limbs, gradual diminution of temperature, extreme dyspnœa: death occurred two months after the commencement of the disease; colliquative diarrhœa having somewhat hastened its termination. The principal, and almost the only morbid appearances were a thick false membrane, poured out from the whole surface of the pericardium, which contained about a pint and a half of a red and turbid serum.

We find, in this case, that the connexion between the disease and its symptoms is so evident, as to render any comment almost unnecessary: the pain corresponded with the parts affected, and depended on the same cause as the palpitations and dyspnœa; the rapidity of their appearance was owing to the sudden accession of the inflammation; the almost constant irregularity of the pulse indicated a very serious lesion of the central organ of the circulation; percussion and auscultation served to confirm a diagnosis, which, even independently of their assistance, might be considered as unquestionable.

The projection of the ribs in the præcordial region forms one of the most peculiar circumstances in this case. Should it be found to exist in other instances, it will become one of the most important diagnostic signs. Here, there was no anormal change in the structure or conformation of the ribs, by which such a circumstance might be explained, and it only corresponds with a similar effect produced by effusion consequent on pleuritis. Its cause would naturally appear to have been the effusion into the pericardium; but this, as well as the reliance which may be placed on it as a sign, can be decided by observation alone.

The decisive character of the symptoms here enumerated, *i. e.* the sudden appearance of violent pain in the præcordial region; palpitations; dyspnœa; irregular pulse, with the signs afforded by the stethoscope and percussion, and these occurring in a subject previously in perfect health, render it difficult to confound this with any other affection of the heart; and an unpardonable negligence alone could account for its being mistaken for any pulmonary affection.

Among the secondary signs in this case, the state of the digestive organs was in perfect harmony with that of those primarily affected. The mucous membrane of the stomach and small intestines was of a red colour, but of healthy consistence and thickness, a condition which it is quite unnecessary to say was not inflammatory; that of the colon was thickened and softened, an inflammatory state which readily explained a diarrhœa which had existed a considerable time previous to death. The distended pericardium might be considered as the cause of cough; but this was more probably dependent on some granulations, which were developed in the summit of the lungs.

The second case, with the exception of a few unimportant varieties, affords a complete analogy with the first. Its subject was a female, aged 47, in whom, after great mental anxiety and bodily fatigue, the affection suddenly commenced by an acute pain in the præcordial region; a sensation of constriction and palpitations. An intermission of three days occurred in the symptoms, after which they returned, with gradually increasing oppression, obliging the patient to observe an almost upright position. On the 26th day of the affection (the first on which she was brought to the hospital), together with these symptoms, the pulsations of the heart were tumultuous and unequal; pulse irregular and often intermittent; chest sonorous in its whole extent, except at the præcordial region, which rendered a dull sound on percussion. Some cough, and crepitation at the lower part of the right lung indicated a slight complication of peripneumony; but while this remained stationary, the symptoms depending on affection of the pericardium went on increasing rapidly. The lower extremities became œdematous, their temperature diminished, and on the 37th day after the attack the patient died, in state of suffocation. The almost only morbid appearances presented by the examination of the body were, a thin and pale membrane, enveloping the whole surface of the heart, and a reddish effusion, of about a pint, into the pericardium.

With the exception of the dilatation of the præcordial region, of which, in this case, I can neither assert nor deny the existence, this example of pericarditis almost exactly resembles the preceding. Its course was somewhat more rapid, but the character of its symptoms was the same, and from this circumstance I am disposed to believe that we may consider as pathognomonic signs of pericarditis—a more or less violent pain in the præcordial region, the access of which is sudden, accompanied by oppression and palpitations, varying in degree; irregular or intermittent pulse; then, after a longer or shorter period, a totally dull sound in the region of the heart, the rest of the thorax rendering a clear sound on percussion. Every time that this assemblage of symptoms presents itself, in a subject *previously quite in health*, we may conclude that pericarditis exists. The absence of pain, provided the other symptoms exist, should cast but little doubt on the diagnosis; for the question can only be between pericarditis and hydropericardium, the latter affection developing itself less rapidly, and without the connexion of symptoms already mentioned. A very chronic form of inflammation of the pericardium would much increase the difficulty of diagnosis. The œdema and diminution of temperature are symptoms common to this affection and diseases of the heart, and they form a line of demarcation between such diseases and affections of other organs. In these two cases, the general correspondence of symptoms was not accompanied by a correspondence in the morbid productions. In the former, the false membrane was thick, formed of two distinct portions effused from the whole surface of the serous membrane; in the latter, the heart alone was covered by a thin coating, interspersed with granulations.

I have seen, besides, five other cases of pericarditis; three of which died so soon after their entrance into the hospital, that no enquiry into the symptoms was made; the fourth was a fatal case of acute ramollissement of the brain, in which palpitations and a very irregular pulse were found to exist with partial pericarditis; the last, a case of phthisis, the pulse having been very irregular for thirty days preceding death, and in which the pericardium was found inflamed. In these instances the want of a careful examination, and not any peculiar difficulty in the diagnosis, probably explains the occurrence of death, without any suspicion having been excited of the existence of inflammation of the pericardium.

There are 36 cases of this disease cited in the works of Morgagni, Corvisart, MM. Bertin, Tacheron, Leroux, and Boyer, among which more than two cannot be selected, in which a proper examination was made. The remainder are incomplete, partly owing to imperfect observation, partly to the circumstance of the patient's not having been seen until a very short time before death, but they are nevertheless useful as confirming the symptoms which I have adduced as characteristic of pericarditis, and as tending to throw light on other points in the history of this disease.

From the cases of Morgagni, none can be collected in which the entire combination of symptoms was observed, and they are consequently almost valueless as regards the establishment of a correct diagnosis. The same objection may be made, with some limitations, to the cases which are contained in the works of Corvisart; (sur les maladies du cœur)—for although he has taken great care, and employed his usual powers of investigation, there are some essential points quite overlooked. Of the eight cases which he has related, four are complications of pericarditis, with pleuro-pneumonia; in three of these there was pain, always on the left side and with one exception, in the præcordial region, although in one instance the pleuro-pneumonia existed on the right side. In another case, in which the pericarditis was uncomplicated, the præcordial region was the seat of pain, in the remaining observations no notice is taken of this symptom. Palpitations are mentioned as having occurred in one case only—irregular, intermittent, and unequal pulse in six—occasional short attacks of faintness in one case—in three, one of which was simple pericarditis, the other two complications with pleuro-pneumonia, the anxiety and sense of suffocation was extreme. In three cases alone of the eight was percussion practised, and in two of these without the requisite care. For instance, in one case of simple pericarditis, the chest having been percussed on the 6th day of the affection, it is said that the whole of the left side gave a dead sound. The assertion that, in a simple case of pericarditis, so large a surface rendered a dead sound, is one which almost carries with it its own contradiction, and it affords an instructive lesson of the importance to be attached to this means of diagnosticating pericarditis (when properly employed), as there can be no doubt that a certain evidence of its existence would have resulted from carefully observing the obvious sources of error.

Some may object to the degree of importance which I attach to the evidence afforded by percussion, from the frequent complication of pericarditis with such affections of the lung as give rise to similar signs. Such an objection must be admitted as tenable whenever there is double pleuritis or pleuro-pneumonia, or if either of them exist on the left side; but, in other cases, the value of percussion is as great as when pericarditis is quite uncomplicated; and that such cases are not of uncommon occurrence may be deduced from the following circumstances, that of seventeen cases of pericarditis complicated with peripneumonia (related by Morgagni, Corvisart, and Bertin), six were of pleuro-pneumonia of the left side, five of both sides, and the remaining six of the right side. Thus, in one-third of these cases, percussion might have been most advantageously employed; but of the 36 cases at present under consideration, twelve are of simple pericarditis, and, by adding these to the previous six, there remains no less than eighteen cases in which percussion might have been advantageously employed.

Another objection to this mode of diagnosis, is founded on the assertion that, in the majority of instances, the quantity of the effusion into the pericardium is insufficient to produce an obscure sound in the præcordial region. Such an objection is not supported by an appeal to facts. Among these 36 cases, the effusion was rarely less than eight or ten ounces, and we must admit that this quantity would suffice to render the sound on percussion obscure, since Corvisart records a case in which only a little fluid was found in the pericardium after death, and where percussion had rendered distinct evidence of its existence. *Pain* is not mentioned as a symptom of universal occurrence. Corvisart and Bertin have not noticed its existence in more than half the cases which they have published, and such would appear to be about its proportion from cases subsequently related.

With regard to the irregular action of the heart, showing itself by irregular pulse, I am disposed to consider it as a valuable symptom of the disease, but that it may be duly appreciated it is necessary to examine the circulation frequently, since it is a condition which may exist without being continuous, and it is probably owing to this circumstance that those who have written on pericarditis have not given to the irregular state of the pulse, that importance as a sign, which it appears to me to deserve.

Fainting is comparatively rare, so much so as not to merit any mention as a symptom. Of the 36 cases, in three only was fainting observed; and it constitutes too alarming an occurrence to suppose that it can have been overlooked.

Palpitations are frequent, though from their comparative insignificance they have not been commonly noticed.

The dyspnœa which exists in all cases cannot be estimated as a sign of any value, since it is common to this and any other thoracic affection; nevertheless, its sudden accession without any evidence of acute pulmonary affection deserves the attention of the physician, and should rouse his suspicions as to whether its cause may not be in a lesion of the pericardium.

Among the remote affections, and one which is common to this and other diseases of the heart, is œdema of the lower extremities—a condition which existed in the two cases mentioned in the commencement of this paper.

From what has been stated, concerning the frequency of the cases in which irregularity of the pulse and pain in the præcordial region co-exist with an obscurity of sound on percussion of the same part, we are justified in concluding that pericarditis may be recognised in about half the cases in which it occurs, and that in many of the simple cases it is as easily recognisable as the best marked example of pleuritis.

It is difficult to state any thing precise as to the duration and progress of pericarditis, but from some facts mentioned by Corvisart and others, taken in conjunction with those at the beginning of this Essay, we are led to the conclusion, that at its onset the disease is generally violent, that the symptoms soon diminish in intensity, and that death takes place between the 24th and 80th day from the commencement. (We shall presently see that to estimate 24 days as the shortest duration of acute pericarditis is not borne out by subsequent investigations.)

The prognosis of pericarditis is a subject of much difficulty. Its complication with other diseases of an equally serious if not more alarming character, accounts for this difficulty. By estimating from the examination of 670 subjects indiscriminately situated, some of which I have personally examined, and the histories of others I have selected from various sources, (in a certain proportion morbid appearances indicating inflammation of the pericardium, such as old adhesions, or the result of more recent inflammations being found to exist,) I have been led to conclude the probable proportion of fatal cases of pericarditis to be one of every six in which this lesion occurs.

Its causes are enveloped in much obscurity. Women are less frequently affected with it than men, and it appears to occur oftener in youth and old age than during the intermediate period of life. There seems to be some ground for the supposition that affections of the heart have some influence in the production of pericarditis, from the comparative frequency in which the morbid effects of this inflammation are found in connexion with other diseases of this viscus. Corvisart relates a case in which it followed a blow, and Bertin in which it appeared subsequently to great exertion. In one of the cases mentioned in this paper it was preceded by fatigue and anxiety. From the frequency of its occurrence, in connexion with pleuro-pneumonia, it is probably excited by similar causes, and may be subject in such cases to its influence.

The cases and remarks contained in this Clinique Medicale of Andral are generally confirmatory of the views contained in the previous part of this paper. In order to avoid prolixity or any useless repetition, we will shortly analyze the nine cases which form the ground-work of Andral's opinions; and, in doing so, we will follow the arrangement of symptoms which Louis has adopted, omitting such as are common to all affections of the heart; and first, with regard to—

*Percussion.* In five only of the nine cases here related was percussion employed, although effusion existed in all excepting one. Of these five one was a suspected case only, the patient having recovered; in three, a death-sound was detected at the præcordial region, and after death considerable effusion was found; in the last, the sound on percussion was normal, and the chief morbid appearance was false membrane lining the pericardium; the quantity of effused fluid not amounting to one ounce. (It is important to remark, that in each case, in which the signs afforded by percussion were so distinct, the inflammation of the pericardium was uncomplicated with any other disease within the thorax.)

*Pain.* Of the nine cases four only were accompanied with pain, one of these being the case which recovered. The pain was not of the same character in all; in the first case, which was of so violent a character as to terminate fatally in 24 hours from its commencement, it was an acute tearing pain; in another instance it extended down the arm, and resembled very much the phenomena of angina pectoris.

*State of the pulse.* In three cases, the state of the pulse is not mentioned; in two it is said to have been regular and remarkably hard; in the remaining four very irregular and intermittent.

*Fainting* is not noticed in any case, although some are examples of the most acute form of the disease. *Palpitations* are not noticed.

*Dyspnœa* is a symptom of universal occurrence. One case is related in which dyspnœa existed as the only symptom.

With regard to the duration of the affection, several cases are mentioned of a far more acute character than those from which M. Louis has drawn his estimate of the average length of the disease. One terminated in 24 hours from its commencement, and three others in less than six days. Of those cases in which pericarditis must be considered as the cause of death, the disease being uncomplicated, four died out of five.

With regard to the causes of the disease, one of comparatively frequent occurrence appears to have escaped M. Louis' notice; *i. e.* metastasis of rheumatic inflammation. The two most severe cases mentioned by Andral followed the subsidence of acute articular rheumatism. One occurred in an advanced stage of phthisis—one in a patient with confluent small-pox; and the rest could be attributed to no cause.

The comparative frequency of the disease affecting one sex mentioned by Louis is confirmed by Andral's cases. Of the nine, one only was a woman; the remainder were young men, mostly between the ages of 20 and 30.

The diagnosis of pericarditis, when complicated with pulmonary or pleuritic inflammation, has not appeared to the authors of the present essays to be worthy of any separate attention, as when they do occur their symptoms are for the most part so unequivocal, as to render the probability of mistake very far from likely to happen, and even should either the one or the other be overlooked, the similarity of the treatment adopted in either case must render such an omission comparatively unimportant. When doubt does exist as to the locality of the thoracic affection, it is by negative evidence, "par voie d'exclusion," that the diagnosis must be determined.

The subject of the stethoscopic signs has also received a very cursory notice, from which we must conclude that the indications which they afford are not of much value—the heart's impulse is sometimes increased, at others jerking, or obscure. What Laennec says on the subject appears to comprise nearly all the information hitherto obtained—"that the ventricular contractions are characterised by a more marked impulse and sound than in the natural state, varying at indefinite intervals with weak and short pulsations, corresponding with the intermissions of the pulse, the weakness of which contrasts strongly with the force of the heart's action." A sound which its discoverers have designated by the term "bruit de cuir," similar to that produced by rubbing two surfaces of leather against each other, has been mentioned as always existing in those cases in which false membranes, or adhesions, are formed between the pericardium and heart, without the co-existence of any effusion. This has been particularly noticed in cases of chronic pericarditis; and however we may be disposed to reject it, at present, as a certain sign of what it is supposed to indicate, it appears to be deserving of careful investigation. Another sign obtained by auscultation, which is said by a late pathologist to be universally present in pericarditis, is the bellows murmur. As the degree of confidence which is to be placed in this phenomenon, as an indication of any pathological condition, is very uncertain, it is difficult to decide how far it may be admissible as an addition to the list of auxiliary symptoms, although, when occurring in combination with other characteristic signs, it may not be without its value.

The subject of treatment of acute pericarditis has received from Louis the attention which might be expected from one who appears to consider almost the whole of medicine as comprised in diagnosis. Very moderate general and local treatment, slight counter-irritation, with abstinence and rest were the means employed in the two cases which are related in the commencement of his essay; and when speaking generally of this subject, he hazards only the following remarks—that from the cases on record nothing decisive on the subject of treatment can be stated; that from the exacerbations of symptoms, which, in the cases which he has related, followed any excitement, whether the most simple motion or the slightest cough, it is evident that absolute rest constitutes one of the most important principles in the treatment of pericarditis.

The later details of Andral, although entering more fully into the means of cure, contain but one example out of the nine cases, in which those means were successful in putting a stop to the disease. From this case he proves the necessity of guarding carefully against too much presumption, in supposing that a considerable alleviation of the symptoms is to be accompanied with any diminution in the activity of treatment, as in this affection more than in any other, he considers that, after such alleviation, a return of acute, or a substitution of chronic inflammation is to be anticipated. The chief means employed, as recommended by Andral, are, copious general and local depletion, with digitalis during the early stage, and subsequently various modes of counter-irritation, together with auxiliary means, which must be evident to every practitioner.

In addition to the pages which in the Clinique Medicale are devoted to acute pericarditis, is a short notice of the disease in the chronic form, a subject which has not received a separate attention from M. Louis. Its symptoms (says Andral) are those chiefly of an organic affection of the heart. We will shortly quote the two cases recorded—the first is that of a mason, aged 25, who in December, 1823, caught a cold, which was accompanied with dyspnœa, and towards the end of January, with œdema of the lower extremities and ascites. At the end of February, his face and lips were livid—there was œdema of the lower limbs—ascites—short and quick respiration—mucous râle over various parts of the chest, which sounded well on percussion—cough and mucous expectoration—heart natural in its impulse, but as well as the pulse, intermittent—never any pain in the præcordial region—tongue and appetite natural—dyspnœa very much increased by taking food—diarrhœa for three months—urine reddish—skin dry. (Blisters to the legs—stimulants, frictions, and fumigations—diuretics.) These means produced a temporary alleviation of the symptoms, which recurred with aggravation on March 5th, when the upper limbs became œdematous—orthopnœa—pulse very small and remarkably irregular—diarrhœa without pain—death on March 10th, quite sudden and without any new symptom or aggravation. After death, the whole pericardium was found to be adherent to the heart by membranous deposits of an inch in thickness, enveloping the heart like a shell—no other particular morbid appearance.

The second is a case of a man, who in April, 1825, became suddenly affected with palpitations, dyspnœa and fever. He was bled several times and improved under this means. Sometime after he applied for relief, his only symptom being extreme frequency of the heart's action, the pulse being regular and of ordinary force, but 140 in a minute; œdema of the lower limbs commencing. (Blisters to the legs, digitalis and diuretics.) Pulse not affected; no increase of urine; vomiting, which necessitated the discontinuance of the medicine. Œdema increased—ascites—infiltration of the face—dyspnœa—pulse as before—effusion into the pleura characterized by the usual symptoms—death in a few days with colliquative diarrhœa. The same membranous deposits were found in this as in the former cases, interspersed however with small, whitish particles, resembling in some places purulent, in others tubercular matter—sero-purulent effusion in the left pleura—tuberculous deposits in old adhesions of the right pleuræ—effusion into the abdomen.

In comparing these two cases, we find, as points of resemblance, 1st. the same morbid change; 2d. the same infiltration, and other symptoms indicating organic affections of the heart, but the onset of the diseases is different; in the second there is an acute stage which is absent in the first; in the first the pulse is intermittent and irregular, in the second it has a frequency which is unusual in organic affections of the heart, and which lead to the diagnosis of pericarditis. But, bare and unsatisfactory as these symptoms are, we must still confess that there are cases of chronic pericarditis in which no symptom gives rise to the suspicion of its existence, and of which examination alone can afford the proof.

A few remarks on the morbid anatomy of pericarditis will conclude our notice of the present essays. *Redness*, a sign which alone constitutes no proof of inflammation, is not universally present, and when it does exist, is always slight, and generally punctuated, covering the pericardium as it were with spots of blood very nearly approximated to one another. In the most acute specimen of pericarditis, the pericardium itself is generally unaltered, or is at most slighly thickened or opaque. *A large quantity of fluid* is the ordinary effect of pericarditis; in one case (mentioned by Corvisart) it amounted to four pints; this fluid is sometimes a limpid, sanguineous serum, or of a deep red colour—sometimes turbid, and mixed with purulent or albuminous flocculi—in others the effused fluid has all the characters of pus—again, it has appeared to be the result of an hæmorrhagic action. The pericardium and heart are ordinarily carpetted (tapissés) with a *false membrane* of various characters. This may exist on one or the other surface, or confined to a spot on either. It is sometimes thin and granular, with an irregular surface, not inappropriately compared to the appearance of a pine-apple—sometimes of an areolar texture of a peculiar character—sometimes resembling the effect which is produced by the separation of two pieces of marble between which some butter has been placed—sometimes not unlike the second stomach of the calf. Filaments, differing in their mode of arrangement, not unfrequently attach the heart to the pericardium. It is probable that these adhesions never occur unless both surfaces be inflamed, and does not the existence of an isolated inflammation of this sort account for those white spots so often observed on the surface of the heart? The most common form of adhesion appears to be where it is general; the firmness of the media of adhesion varies with a variety of causes.

Chronic pericarditis always occupies the whole membrane, which is generally much more red than in the acute form of the disease. The two cases given by Andral afford examples of what is the effect of this character of inflammation. A tubercular deposition, as in analogous cases in other serous membranes may take place in the substances effused by chronic inflammation. There are also several cases related in which ossific deposits were found both beneath the serous membrane and in the substance of the membranous matter effused by chronic inflammation.

